# Retinoids Regulate the Formation and Degradation of Gap Junctions in Androgen-Responsive Human Prostate Cancer Cells

**DOI:** 10.1371/journal.pone.0032846

**Published:** 2012-04-13

**Authors:** Linda Kelsey, Parul Katoch, Kristen E. Johnson, Surinder K. Batra, Parmender P. Mehta

**Affiliations:** Department of Biochemistry and Molecular Biology, University of Nebraska Medical Center, Omaha, Nebraska, United States of America; University of Kentucky College of Medicine, United States of America

## Abstract

The retinoids, the natural or synthetic derivatives of Vitamin A (retinol), are essential for the normal development of prostate and have been shown to modulate prostate cancer progression in vivo as well as to modulate growth of several prostate cancer cell lines. 9-cis-retinoic acid and all-trans-retinoic acid are the two most important metabolites of retinol. Gap junctions, formed of proteins called connexins, are ensembles of intercellular channels that permit the exchange of small growth regulatory molecules between adjoining cells. Gap junctional communication is instrumental in the control of cell growth. We examined the effect of 9-cis-retinoic acid and all-trans retinoic acid on the formation and degradation of gap junctions as well as on junctional communication in an androgen-responsive prostate cancer cell line, LNCaP, which expressed retrovirally introduced connexin32, a connexin expressed by the luminal cells and well-differentiated cells of prostate tumors. Our results showed that 9-cis-retinoic acid and all-trans retinoic acid enhanced the assembly of connexin32 into gap junctions. Our results further showed that 9-cis-retinoic acid and all-trans-retinoic acid prevented androgen-regulated degradation of gap junctions, post-translationally, independent of androgen receptor mediated signaling. Finally, our findings showed that formation of gap junctions sensitized connexin32-expressing LNCaP cells to the growth modifying effects of 9-cis-retinoic acid, all-trans-retinoic acid and androgens. Thus, the effects of retinoids and androgens on growth and the formation and degradation of gap junctions and their function might be related to their ability to modulate prostate growth and cancer.

## Introduction

Retinoids, the natural or synthetic derivatives of vitamin A, regulate not only embryonic development but also organogenesis in adult tissues [1]. A requirement for vitamin A for proliferation, differentiation has been demonstrated in many studies in which a deficiency of this vitamin resulted in multiple developmental defects [1]–[3]. All trans-retinoic acid (ATRA) and 9-Cis-Retinoic Acid (9-CRA) are the two most important metabolites of vitamin A (retinol) with diverse physiological functions [1]. Retinoids are members of the nuclear-receptor superfamily of transcription factors and exert their pleiotropic effects by regulating the expression of several target genes [3]–[5]. There are six retinoid receptors, namely RAR α, β, γ, which bind to ATRA and 9-CRA, and RXR α, β, γ, which bind only to 9-CRA. Retinoid initiated signaling regulates several homeostatic control mechanisms during embryonic development and in adult tissues and one such control mechanism likely to be regulated is the direct cell-cell communication mediated by a special class of cell junctions called gap junctions (GJs) [6]–[8]. Gap junctions are ensembles of intercellular channels that signal by permitting the direct exchange of small molecules (≤1500 Da) between contiguous cells. The constituent proteins of GJs, called connexins (Cxs), are coded by 21 genes, which have been designated according to their molecular mass [9]. Cell-cell channels are bicellular structures formed by the collaborative effort of two cells. To form a GJ cell-cell channel, Cxs first oligomerize as hexamers, called connexons, which dock with the connexons displayed on contiguous cells [10]. Multiple lines of evidence lends credence to the notion that gap junctional communication is an important homeostatic control mechanism for regulating cell growth and differentiation. For example, impaired Cx expression, or loss of function, has been implicated in the pathogenesis of several types of cancers, and mutations in several Cx genes have been detected in genetic disorders characterized by aberrant cellular proliferation and differentiation [8], [10]–[12]

Our previous studies showed that prostate luminal cells expressed Cx32 and its expression coincided with the acquisition of the differentiated state of these cells [13], [14]. We showed that progression of prostate cancer (PCA) from an androgen-dependent state to an invasive, androgen-independent state was characterized by the failure of Cx32 to assemble into GJs [14], [15]. We have further shown that reintroduction of Cx32 into androgen-responsive human PCA cell line, LNCaP, retards cell growth *in vivo* and *in vitro* [14], [16]. Subsequently, we demonstrated that androgens regulated the formation and degradation of GJs by altering the expression level of Cx32, posttranslationally. In the absence of androgens, a major fraction of Cx32 was degraded by endoplasmic reticulum associated degradation (ERAD) whereas in their presence this fraction was rescued from degradation [17]. The significance of these findings is underscored by the fact that androgens play a major role in the survival and maintenance of the secretory (differentiation-related) function of luminal epithelial cells of normal prostate as well as of tumor cells as androgen ablation induces apoptosis or dedifferentiation of these cells [18], [19]

Like androgens, retinoids are also essential for the normal development of the prostate and modulate PCA progression in certain mouse models as well as suppress the growth of androgen-dependent and -independent human PCA cell lines. Squamous metaplasia of the prostate was observed among the offsprings of vitamin A-deficient [20] and RARγ knock out mice [21]. Moreover, tissue-specific inactivation of RARα in prostate resulted in multi-focal intraepithelial hyperplasia [22]. Epidemiological studies have shown that decreased vitamin A serum levels increase PCA incidence and progression, and that restoration of retinoid levels might have a role in the reversal of the malignant phenotype [23], [24]. Several studies, including ours, have shown that the ability of retinoids to suppress tumor cell growth and induce differentiation is contingent upon their ability to enhance gap junctional communication [25]–[28]. Because Cx32 is expressed by luminal epithelial cells of normal prostate where it is assembled into GJs and by epithelial cells of prostate tumors in which it is inefficiently assembled into GJs [13]–[15] and because formation of GJs has been implicated in maintaining the polarized and differentiated state of epithelial cells [29], we reasoned that chemopreventive, growth inhibitory and pro-differentiating effects of 9-CRA and ATRA might result from their ability to control formation and degradation of GJs. Therefore, we investigated whether the formation and degradation of GJs composed of Cx32 were regulated by 9-CRA and ATRA in human PCA cells. Because retinoids have been shown to increase the expression of androgen receptor (AR) in androgen-responsive human PCA cell lines [30], we rationalized that they might modulate androgen-regulated formation and degradation of GJs and affect growth of androgen-responsive PCA cells that express Cx32. By using androgen-responsive LNCaP cells, which express retrovirally introduced Cx32 whose degradation is androgen-regulated [17], we show that 9-CRA and ATRA, like androgens, enhance the expression of Cx32 and its subsequent assembly into GJs. Moreover, we further show here that in this cell culture model, ATRA and 9-CRA prevent androgen-regulated degradation of GJs, independent of AR mediated signaling. Finally, our findings show that expression of Cx32 and formation of GJs sensitizes these cells to growth modifying effects of 9-CRA, ATRA and androgens.

## Materials and Methods

### Cell Culture

Androgen-responsive LNCaP cells were a gift from Dr. Lin [31]. One of the several clones of LNCaP cells expressing retrovirally transduced rat Cx32, hereafter referred to as LNCaP-32 cells, and one of the several control clones selected in G418 after infection with control retrovirus, hereafter referred to as LNCaP-N cells, were isolated as described [17] and were used in the present study along with the parental LNCaP cells, hereafter referred to as LNCaP-P cells. LNCaP-P cells were grown in RPMI containing 5% fetal bovine serum in an atmosphere of 5% CO2/95% air and stock cultures were maintained weekly as previously described. LNCaP-N and LNCaP-32 cells were maintained in RPMI containing 5% fetal bovine serum and G418 at 200 µg/ml as describe [17]. During the course of these studies, we used two separate lots of fetal bovine sera obtained from Sigma and HyClone Laboratories, with nearly similar effect on the growth of LNCaP cells. Steroid-depleted (charcoal-stripped) serum was obtained from HyClone Laboratories (Salt Lake City, UT). We also used phenol red free RPMI for experiments in which charcoal-stripped serum was used [17].

### Antibodies and Immunostaining

Hybridoma M12.13 (a gift from Dr. Dan Goodenough, Harvard University) has been described earlier [14], [16], [17], [32], [33]. Mouse anti-occludin (clone OC-3F10) was from Zymed laboratories Inc. (South San Francisco, CA). Rabbit anti-α-catenin, rabbit anti-β-catenin, rabbit anti-Cx32, and mouse anti-β-actin (clone C-15) were from Sigma (St. Louis, MO). Mouse anti-E-cadherin, mouse anti-α-catenin, mouse anti-β-catenin antibodies were generously provided by Drs. Johnson and Wheelock (Eppley Institute) and have been described [17], [32], [33]. A rabbit polyclonal anti-AR receptor antibody was from Santa Cruz Biotech (sc-13062, San Diego, CA). Cells were immunostained after fixing with 2% para-formaldehyde for 15 min as described previously [17], [32], [33]. Briefly, cells (1.5×10^5^), seeded in six well clusters containing glass cover slips and allowed to grow to approximately 50–70% confluence, were immunostained at room temperature with various antibodies at appropriately calibrated dilutions. Secondary antibodies (rabbit or mouse) conjugated with Alexa 488 and Alexa 594 were used as appropriate. Images of immunostained cells were acquired with Leica DMRIE microscope (Leica Microsystems, Wetzler, Germany) equipped with Hamamatsu ORCA-ER CCD camera (Hamamatsu-City, Japan). For co-localization studies, serial z-sections (0.5 µm) were collected and analyzed using image processing software (Volocity; Improvision, Inc; Perkin Elmer).

### Stock Solutions

Stock solutions of various reagents were prepared as follows: 9-CRA and ATRA (BIOMOL, Plymouth, PA) were prepared in ethanol at 3 mM each and stored in aliquots at −80°C protected from light. All experiments pertaining to 9-CRA and ATRA were performed in yellow light as described [26], [27]. Stock solutions of a synthetic androgen, mibolerone (MB), (BIOMOL), and of a natural androgen, dihydro-testosterone (DHT), were prepared at 1 mM in ethanol and stored at −20°C in small aliquots protected from light. They were appropriately diluted with the medium at the time of treatment.

### Androgen Depletion and Other Treatments

Cells were seeded in six well clusters containing glass cover slips (1.5×10^5^ cells per well) and in 6-cm (2×10^5^ cells per dish) or in10-cm dishes (3.5×10^5^ cells per dish) in 2, 4 and 10 ml complete culture medium, respectively. Cells were treated when approximately 50% confluent, by replenishing with fresh medium containing various reagents at the desired concentration added from stock solutions so that the final concentration of the solvent did not exceed 0.3%. To grow cells under androgen-depleted medium, normal cell culture medium was replaced with androgen-depleted cell culture medium (phenol red-free RPMI containing 5% charcoal-stripped serum). The controls received fresh medium containing normal serum.

### Western Blot Analysis and Detergent Solubility of Connexin32

Cell lysis, detergent solubility assay with 1% Triton X-100 (TX-100) and the expression level of Cx32 were analyzed by Western blot analysis as described [17], [32], [33]. Briefly, 5×10^5^ LNCaP-P, LNCaP-N and LNCaP-32 cells were seeded per 10 cm dish in 10 ml of complete medium and grown to confluence, after which cells were lysed in buffer SSK (10 mM Tris, 1 mM EGTA, 1 mM PMSF, 10 mM NaF, 10 mM NEM, 10 mM Na_2_VO_4_, 10 mM iodoacetamide, 0.5% TX-100, pH 7.4) supplemented with the protease inhibitor cocktail (Sigma, St. Louis, MO). Total, detergent-soluble and -insoluble extracts were separated by ultracentrifugation at 100,000×g for 60 min (35,000 rpm in analytical Beckman ultracentrifuge; Model 17–65 using a SW50.1 rotor). The detergent-insoluble pellets were dissolved in buffer C (70 mM Tris/HCl, pH 6.8, 8 M urea, 10 mM NEM, 10 mM iodoacetamide, 2.5% SDS, and 0.1 M DTT). Following normalization based on cell number, the total, TX-100-soluble and -insoluble fractions were mixed with 4× SDS-loading buffer to a final concentration of 1× and incubated at room temperature for 1 h (for Cx32) before SDS-PAGE analysis.

### Communication Assays

Gap junctional communication was assayed by microinjecting the following fluorescent tracers: Lucifer Yellow (MW 443 Da; Lithium salt); Alexa Fluor 488 (MW 570 Da; A-10436), and Alexa Fluor 594 (MW 760 Da; A-10438). Stock solutions of Alexa dyes, obtained as hydrazide sodium salts from Molecular Probes (Carlsbad, CA), were prepared in water at 10 mM. Lucifer yellow was microinjected as 2.5% aqueous stock solution. Eppendorf InjectMan and FemtoJet microinjection systems (models 5271 and 5242, Brinkmann Instrument, Inc. Westbury, NY), mounted on Leica DMIRE2 microscope as described previously, were used to microinject fluorescent tracers. The images of microinjected cells were captured with the aid of CCD camera (Retiga 2000R, FAST 1394) using QCapture (British Columbia, Canada) and stored as TIFF files. Junctional transfer of fluorescent tracer was quantitated by scoring the number of fluorescent cells (excluding the injected one) from the captured TIFF images either at 1 min (Lucifer Yellow), 3 min (Alexa 488) and 15 min ( Alexa 594) after microinjection into test cell as described [14], [17], [32], [34].

### Colony Formation and Cell Growth Assays

Cell growth was assessed either by colony forming assay or by counting cells as described [14], [34]. For colony forming assay, 2×10^3^ cells were seeded in 6 cm dishes in triplicate in 3 ml culture medium. After 24 h, one ml medium containing MB, 9-CRA and ATRA was added to the dishes to give the desired final concentration. Cells were grown for 3–4 weeks (with a medium change every 4–5 days containing the appropriate concentration of MB, DHT, 9-CRA and ATRA) when they formed visible colonies. Colonies in dishes were fixed with 3.7% buffered formaldehyde, stained with 0.025% solution of crystal violet in PBS, and photographed. For measuring cell growth, 5×10^4^ cells were seeded in 6 cm dishes in replicate and treated with MB, 9-CRA and ATRA either alone or in combination as described above. Cells were allowed to grow for 9–11 days with a medium change at day 5. Cells were trypsinized and counted in a hemocytometer.

## Results

### Retinoids Enhance Cx32 Expression Level

**Figure 1:**
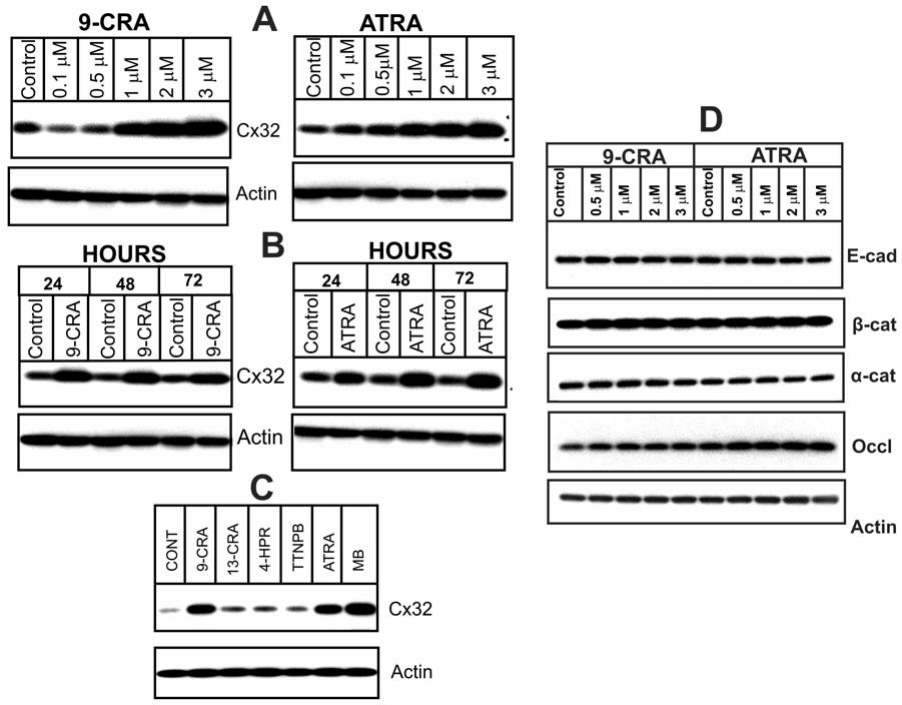
Retinoids increase Cx32 expression level. Cx32-expressing LNCaP-32 cells were treated with the 9-CRA, ATRA, MB and other retinoids as indicated. **A**. Dose-dependent enhancement of Cx32 expression level upon 9-CRA and ATRA treatment for 48 h. Note that significant enhancement is observed only at concentrations above 0.5 µM. **B**. Kinetics of enhancement of Cx32 expression level upon treatment with 9-CRA and ATRA (1 µM) for the indicated times. Note that enhancement is observed as early as 24 h. **C**. Only 9-CRA and ATRA and MB increase Cx32 expression level. Note that RAR-specific retinoids, TTNPB (tetrahydrotetramethyl-nepthalenylpropanylbenzoic acid), and 13-CRA (13-cis-retinoic acid) and the other unrelated retinoid,4-HPR, are ineffective. Note that MB (2.5 nM) is at least 300–500 times more potent than 9-CRA and ATRA on equimolar basis. **D**. Effect of 9-CRA and ATRA on adherens and tight junction associated proteins. Expression of adherens junction associated proteins E-cadherin and α- and β-catenins, and tight junction associated protein, occludin, was analyzed by Western blot analysis of total cell lysate (5 µg). Note that only the expression of occludin appears to change noticeably.

We used LNCaP-32 cells that express retrovirally transduced rat Cx32 [17] because parental LNCaP-P and LNCaP-N cells are devoid of detectable levels of known Cxs and do not form functional GJs (See Materials and Methods). Earlier studies showed that in LNCaP-32 cells androgens regulated the formation and degradation of GJs by controlling the expression level of Cx32 posttranslationally, by rescuing its ERAD-mediated degradation [17]. Based on the rationale described above (see introduction) and on our experience with other cell lines [26], [27], LNCaP-32 cells were treated with 9-CRA and ATRA for 48 h to examine if they affect Cx32 expression level. We found that 9-CRA and ATRA increased Cx32 expression level in a dose-dependent manner (Figure 1, A). Significant enhancement, however, was observed only at concentration of 1 µM or higher. As assessed by the colony formation assay, concentrations higher than 1 µM were toxic to these cells (data not shown) and hence we chose 1 µM of 9-CRA and ATRA for subsequent studies. Time course studies showed that enhancement with both retinoids occurred as early as 24 h post-treatment and reached a plateau at 72 h (Figure 1, B). The effect of 9-CRA and ATRA on Cx32 expression level was comparable to that observed with a synthetic androgen, mibolerone (MB), and was not observed with RAR-specific ligands, 13-CRA and tetrahydrotetramethyl-nepthalenylpropanylbenzoic acid (TTNPB), or with 4-hydroxy-phenretinamide (4-HPR)(Figure 1 C). We also examined whether 9-CRA and ATRA increased the expression level of other cell junction associated proteins. We found that both 9-CRA and ATRA had no significant affect on the expression level of adherens junction proteins E-cadherin and α- and β-catenins, however, the expression level of tight junction associated protein, occludin, was enhanced to some extent (Figure 1D). Moreover, 9-CRA and ATRA neither induced the expression of endogenous Cx32 in parental LNCaP cells nor altered the expression level of retrovirally transcribed Cx32 mRNA in LNCaP-32 cells as measured by semi-quantitative RT-PCR analysis (data not shown).

### Retinoids Enhance Gap Junction Assembly and Junctional Communication

**Figure 2:**
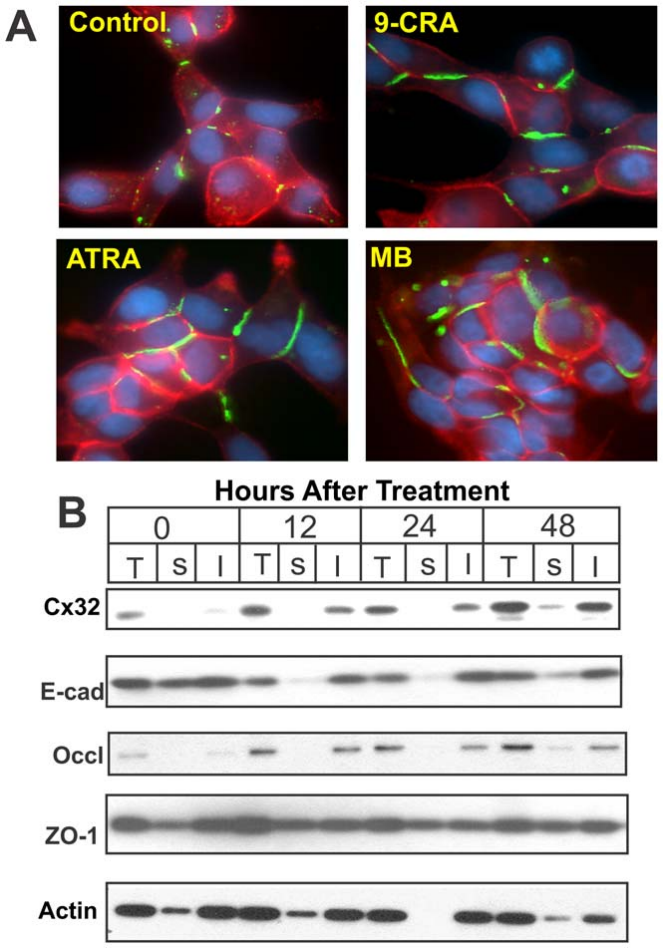
Retinoids enhance the assembly of Cx32 into gap junctions. **A**. LNCaP-32 cells, grown either in six well clusters or 10-cm dishes, were treated with 9-CRA and ATRA for various times. Assembly of Cx32 (green) into GJs was assessed immunocytochemically. E-cadherin is shown in red. Note that GJ formation was enhanced with 9-CRA and ATRA and MB treatment. **B**. Assembly of Cx32 into GJs, of tight junction associated protein, occludin and ZO-1, and adherens junction protein, E-cadherin, was assessed biochemically by TX100 insolubility assay as described in Materials and Methods. Note also that both the total level and the detergent-insoluble fraction of Cx32 increased significantly. Note also that the detergent-solubility of adherens junction associated proteins, E-cadherin, is not significantly affected whereas that of tight junction associated protein, occludin, is marginally enhanced. In (A), the nuclei (blue) are stained with DAPI.

We next examined the effect of 9-CRA and ATRA on the assembly of Cx32 into GJs and on junctional communication. Concomitant with an increase in the expression level of Cx32, 9-CRA and ATRA increased GJ assembly as assessed immunocytochemically (Figure 2 A) and biochemically by Western blot analysis of total and Triton X (TX)-100-insoluble extracts [16], [17], [35] prepared at various times after treatment (Figure 2 B). Moreover, as shown in Table 1, enhancement of GJ assembly was accompanied by parallel increase in junctional communication as measured by the junctional transfer of three GJ permeable fluorescent tracers, Lucifer Yellow (MW 443), Alexa 488 (MW 570), and Alexa 594 (MW 760). For example, 9-CRA and ATRA increased junctional transfer of Alexa 594 (MW 760) 2–3 folds compared to controls (Table 1). Because E-cadherin has been shown to facilitate the assembly of Cxs into GJs [32], [33], and Cx expression has been shown to facilitate the assembly of tight junctions [29], we also examined whether 9-CRA and ATRA increased the expression level of Cx32 and its assembly into GJs indirectly by facilitating the assembly of other cell junctions as assessed by the detergent-insolubility of their constituent and associated proteins. We found that both 9-CRA and ATRA had no significant affect on the expression level of adherens junction protein E-cadherin, however, the assembly of tight junction protein, occludin, but not its associated protein ZO-1, appeared to have been enhanced (Figure 2 B). Taken together, these data suggest that 9-CRA and ATRA, like androgens, enhance the expression level of Cx32, and its subsequent assembly into GJs, without significantly altering the expression of E-cadherin, which has been shown to modulate the assembly of GJs [32], [33], [36], [37]. As was observed in our earlier studies with androgens, the assembly of Cx32 and occludin into cell junctions, or vice versa, appears to be regulated coordinately [17], [29], [38], [39].

**Table 1:** Effect of 9-CRA, ATRA, and androgen on junctional transfer in LNCaP-32 cells.

Junctional Tracer	Expt #	Junctional Transfer^a^			
		NS	NS+9-CRA^b^	NS+ATRA^b^	NS+MB^b^
Lucifer Yellow	1	14.5±4.1(14)	34.4±6.3(18)	24.7±6.5(19)c	32±6.3.7(22)
	2	16.1±3.6(17)	28.7±4.5(14)	22.1±5.1(17)	38.9±7.1(20)
Alexa-488	1	12.8±4.2(19)	24.3±4.7(25)	22.5.±5.2(29)	30.7±7.1(22)
	2	10.2±3.3 (21)	25.8±2.3(22)	20.2±4.8(26)	29.8±8.2(27)
Alexa-594	1	7.7±2.3(25)	17.1±2.9(25)	14.9±3.7(25)	14.1±3.9(27)
	2	5.3±1.7(20)	14.8±4.1(22)	13.3±3.9(22)	15.1±5.7 (22)

LNCaP-32 cells, seeded in 6 cm dishes, were grown to 70% confluence. Junctional transfer was measured after microinjecting fluorescent tracers as described in Materials and Methods.

a : The number of fluorescent cell neighbors (mean ± SE) 1 min (Lucifer Yellow), 3 min (Alexa-488) and 15 min ( Alexa-594) after microinjection into test cell. The total number of injection trials is shown in parentheses.

b : Cells were treated for 48 h with 9-CRA and ATRA (1 µM) and MB (2.5 nM).

### Retinoids Modulate Androgen-regulated Formation and Degradation of Gap Junctions

**Figure 3:**
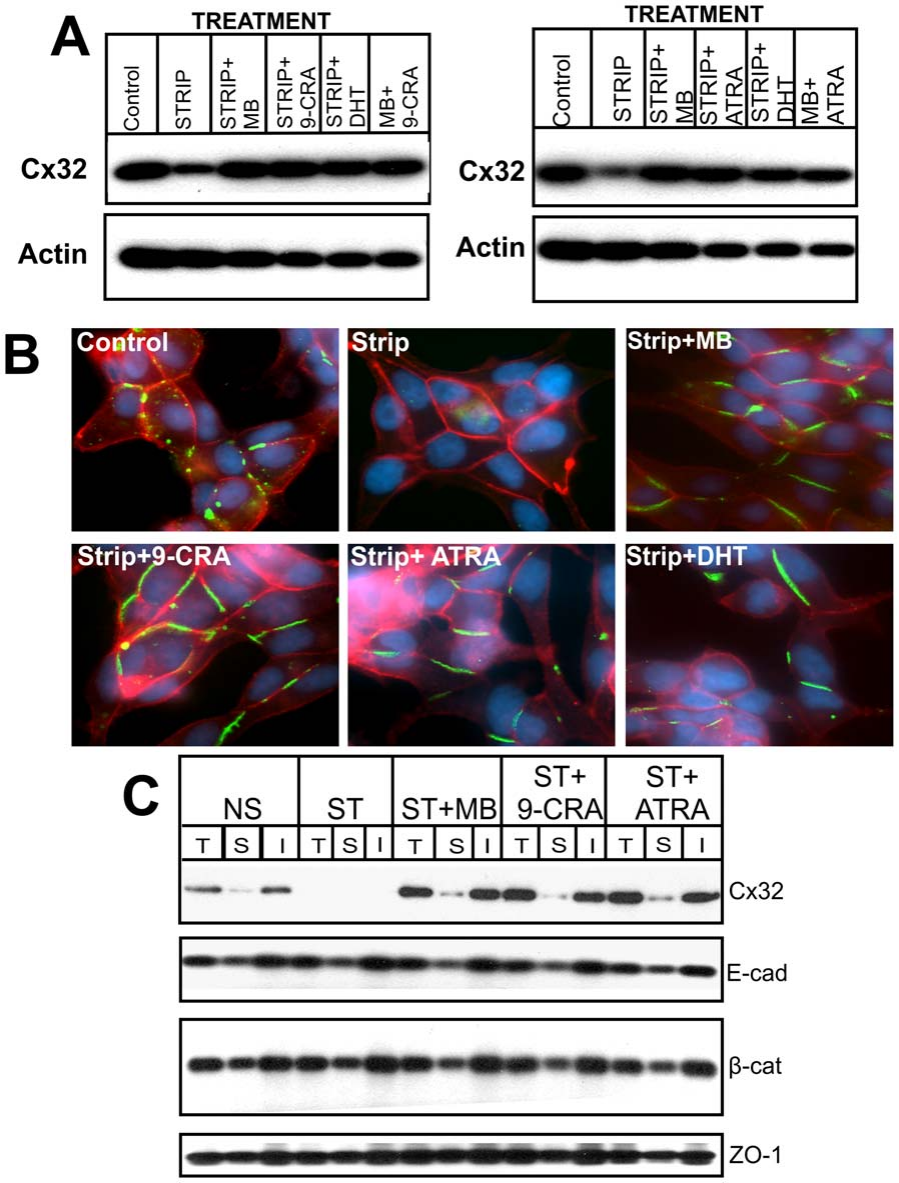
Retinoids block androgen-regulated formation and degradation of gap junctions. LNCaP-32 cells, grown to 70% confluence either in six well clusters or 10-cm dishes, were switched to charcoal-stripped, androgen-depleted (Strip) medium. Expression level of Cx32 and formation of GJs were analyzed by Western blot (**A**) and immunocytochemical analysis (**B**) after 48 h in the presence and absence of 9-CRA and ATRA (1 µM) and MB (2.5 nM) and DHT (10 nM). **C**. Formation of GJs was also analyzed by Western blot analysis of total, detergent-soluble and -insoluble fractions as described in Materials and Methods. Note that Cx32 and GJs are degraded in androgen-depleted medium and degradation is blocked upon replenishment with 9-CRA, ATRA, MB and DHT. Note also that only the expression level of Cx32 and its detergent solubility changed significantly whereas that of E-cadhiern (E-cad), β-catenin (β-cat) and ZO-1 was not significantly affected. In (B), the nuclei (green) were stained with DAPI. In A, 10 µg of total protein was analyzed.

Our previous studies showed that Cx32 was degraded upon androgen depletion by ERAD, and that androgens enhanced GJ formation by re-routing the ERAD-targeted pool of Cx32 to the cell surface [17]. Prompted by the data shown in Figures 1 and 2, we next examined the expression level of Cx32 and its junctional and non-junctional fate upon androgen depletion in the presence and absence of 9-CRA and ATRA. For these studies, cells were grown in androgen-depleted (charcoal-stripped), phenol-red free cell culture medium. Consistent with earlier studies [17], we found that androgen depletion decreased Cx32 expression level, which was prevented upon addition of MB and DHT (Figure 3 A). However, we found that the decrease in the expression level of Cx32 was also prevented when androgen-depleted medium was replenished with 9-CRA and ATRA (Figure 3 A). Moreover, combined treatment with MB and 9-CRA or ATRA was neither synergistic nor additive with respect to Cx32 expression level (Figure 3 A). To substantiate the above data, we assessed the formation of GJs immunocytochemically (Figure 3 B), biochemically by detergent insolubility assay (Figure 3 C), and functionally by measuring the junctional transfer of Lucifer Yellow (MW 443 Da), Alexa 488 (MW 570 Da), and Alexa 594 (MW 760 Da) (Table 2). As shown in Figure 3B, androgen depletion reduced the number of GJs drastically as Cx32-specific immunostaining was rarely observed at cell-cell contact areas, while GJs were readily detected in cells when androgen-depleted medium was supplemented with 9-CRA and ATRA and with MB and DHT (Figure 3 B). Consistent with the immunocytochemical data, junctional transfer of Lucifer Yellow decreased significantly upon androgen depletion, which was prevented upon replenishing androgen-depleted medium with MB, ATRA and 9-CRA (Table 2). The detergent insolubility assay further corroborated the immunocytochemical and junctional transfer data (Figure 3 C). As was observed in our earlier studies, depletion of androgens had no significant effect on the detergent solubility of E-cadherin and β-catenin and tight junction associated protein, ZO-1 (Figure 3 C). As a control, depletion or addition of androgens, 9-CRA and ATRA to parental LNCaP-P and G418-resistant LNCaP-N cells neither induced GJ assembly nor had any effect on the junctional transfer (data not shown). Collectively, these data suggest that 9-CRA and ATRA prevent androgen-regulated degradation of Cx32 and enhance GJ formation in LNCaP-32 cells. Because combined treatment with androgens and retinoids was neither synergistic nor additive, the data further suggest that GJ formation is enhanced by rescuing the same pool of Cx32 which is targeted for ERAD upon androgen depletion.

**Table 2:** Effect of 9-CRA, ATRA and androgen on junctional transfer in LNCaP-32 cells under androgen-depleted conditions.

Treatment	Exp #	Junctional Tracer		
		LY	Alexa488	Alexa-594
NS	1	14.5±4.1(21)	27.4±4.7(24)	15.4±3.3(22)
	2	16.1±3.6(27)	24.8±5.2(27)	16.1±6.1(18)
Strip	1	2.4±0.7(24)	2.1±0.6(24)	0(11)
	2	1.8±0.6.(22)	2.8±0.6.(27)	0(13)
Strip+MB	1	29.4±.5.7(32)	26±4.1(21)	13.3±2.47(23)
	2	32.9±6.3(28)	30.1±5.2(20)	14.4±3.1(19)
Strip+9-CRA	1	27.4±5.1(24)	29.2±8.1(21)	12.5±2.1(22)
	2	33.8±7.4(28)	34.9±6.1(25)	15.9±4.1(18)
Strip+ATRA	1	22.4±3.7(23)	23.7±4.6(24)	13.2±3.3(32)
	2	28.2±4.1(29)	28.9±5.7(24)	15..9±7.1(30)

LNCaP-32 cells, seeded in 6 cm dishes, were grown to 70% confluence, after which they were switched to charcoal-stripped, androgen-depleted medium (Strip) for 48 h in the presence and absence of 9-CRA, ATRA, and MB. Junctional transfer was quantified as described in Table 1 legend and in Materials and Methods.

### Retinoids Enhance Gap Junction Formation Independent of Androgen Receptor Function

**Figure 4:**
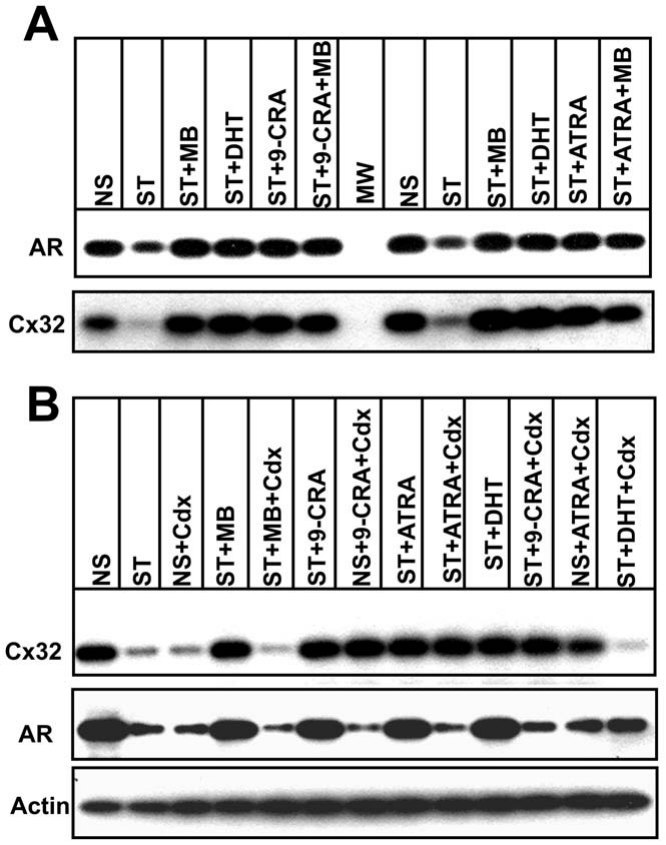
Effect of 9-CRA, ATRA and androgens on the expression level of Cx32 and AR. **A**. LNCaP-32 cells were grown to 70–80% confluence in 6 cm dishes. Cells were switched to charcoal-stripped, androgen-depleted medium (ST) containing 9-CRA and ATRA (1 µM) and MB (2.5 nM) and DHT (10 nM) for 24 h. Cells in androgen-containing medium (NS) or androgen-depleted medium (ST) were used as controls. Expression level of Cx32 and AR was analyzed by Western blotting. Note that the expression level of both Cx32 and AR decreases upon androgen depletion and the decrease is blocked upon treatment with 9-CRA, ATRA, MB and DHT. **B**. LNCaP-32 cells, seeded as above, were treated with of 9-CRA and ATRA (1 µM) and MB (2.5 nM) and DHT (10 nM) in the presence and absence of Casodex (10 µM) in normal and androgen-depleted medium. Expression level of Cx32 and AR was examined after Western blotting. Note that in Casodex containing medium, the expression level Cx32 does not decrease in the presence of 9-CRA and ATRA (1 µM) in charcoal-stripped medium despite low level of AR expression. This is not observed with MB and DHT.

**Figure 5:**
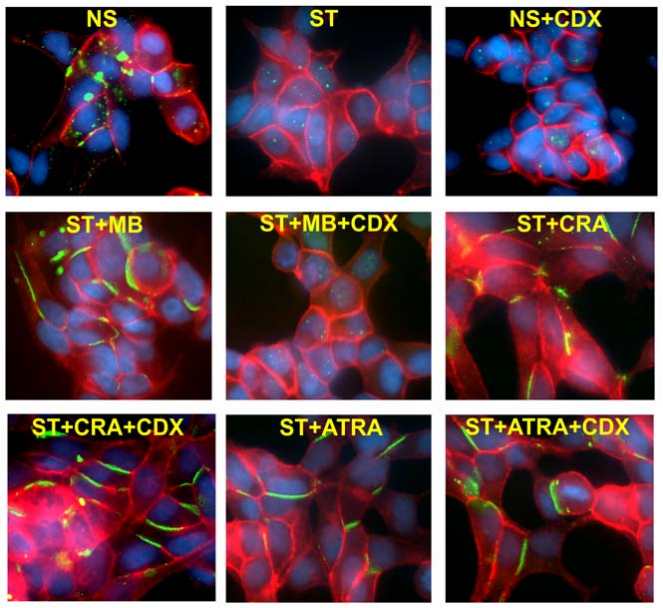
Effect of 9-CRA, ATRA, and androgens on the formation of gap junctions in the presence and the absence of casodex. LNCaP-32 cells, seeded in six well clusters containing glass cover slips, were allowed to grow to 70% confluence. Cells were then grown for additional 24 h in normal medium (NS), in charcoal-stripped, androgen-depleted medium alone (ST), in normal serum containing casodex (NS+CDX), and in androgen-depleted medium supplemented with MB (ST+MB), with MB and casodex (ST+MB+CDX), with 9-CRA (ST+CRA), 9-CRA and CDX (ST+CRA+CDX), with ATRA (ST+ATRA), and with ATRA and CDX (ST+ATRA+CDX). Degradation and subcellular localization of Cx32 were analyzed immunocytochemically as described in Materials and Methods. Note that GJs (green) are not degraded in cells treated with 9-CRA and ATRA both in the presence and absence of casodex whereas they are degraded in normal serum and androgen-depleted but MB supplemented medium containing casodex. E-cadherin is shown in red and the nuclei (blue) were stained with DAPI.

Treatment of LNCaP cells with 9-CRA has been shown to enhance AR expression level [30]. To test if 9-CRA and ATRA enhanced AR expression in normal and androgen-depleted medium, we grew LNCaP-32 cells in control, androgen-depleted, and androgen-depleted plus 9-CRA, ATRA and MB containing medium for 48 h. As assessed by Western blot analysis, we found that androgen depletion reduced both the expression level of AR and Cx32, which was prevented upon replenishment with MB, DHT, 9-CRA and ATRA (Figure 4 A). These data raised the possibility that 9-CRA and ATRA enhanced GJ assembly in an AR-dependent manner — and not independently. To test this notion, we treated LNCaP-32 cells with Casodex (Bicalutamide), which blocks androgen action by competing with androgens for binding to the AR and inhibiting function [40], [41], and examined the expression level of Cx32 and GJ formation upon treatment with 9-CRA, ATRA and MB in the presence and absence of Casodex in normal and androgen-depleted medium. Consistent with previous studies, we found that treatment with Casodex caused degradation of AR (Figure 4 B) as well as abolished the effect of MB and DHT on Cx32 expression as was observed in our earlier studies [17]. However, degradation of AR was also observed with Casodex in the presence of 9-CRA and ATRA both in androgen-depleted and normal medium. Despite robustly decreasing AR expression level, we found that Casodex had no effect on 9-CRA- and ATRA-mediated enhancement of Cx32 expression level. To substantiate that decrease and increase in Cx32 expression was accompanied by parallel changes in GJ formation, we examined the formation of GJs immunocytochemically in Casodx-treated cells in the presence and absence of MB, 9-CRA and ATRA. GJs were not formed when cells were treated with casodex in normal serum or in androgen-depleted medium containing MB or DHT (Figure 5). On the other hand, we found that GJs were abundant when cells were treated with casodex along with 9-CRA or ATRA (Figure 5). These data suggest that the mechanism by which 9-CRA and ATRA prevent the degradation of Cx32 and enhance GJ formation upon androgen depletion is independent of AR expression level and/or function or both.

### Connexin32 Expression Alters the Response of LNCaP Cells to Growth Modulatory Effect of Retinoids and Androgens

**Figure 6:**
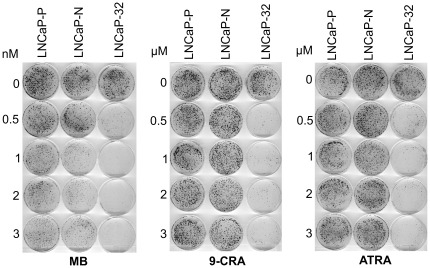
Connexin expression and junction formation accentuates the growth inhibitory effect of 9-CRA, ATRA and androgens in LNCaP cells. LNCaP-P, LNCaP-N and LNCaP-32 cells were seeded at a clonal density in 6 cm dishes in triplicate (2×10^3^ cells per dish). After 24 h, cells were treated with the indicated concentrations of 9-CRA, ATRA and MB. Cells were grown until they formed visible colonies (21 d for LNCaP-P and LNCaP-N and 33 d for LNCaP-32). Medium was changed every 4 d. Colonies were fixed with formalin and stained with crystal violet as described in Materials and Methods. Note that the growth of LNCaP-32 cells is profoundly inhibited upon 9-CRA, ATRA and MB treatment as colonies are barely detectable.

**Figure 7:**
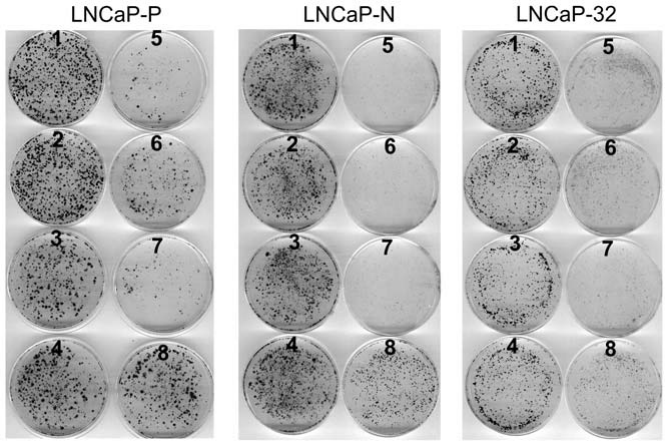
Connexin expression and junction formation accentuates the growth inhibitory effect of 9-CRA, ATRA and androgens in LNCaP cells. LNCaP-P, LNCAP-N and LNCaP-32 cells were seeded at clonal density in 6 cm dishes in triplicate as described in Figure 6 legend. After 24 h, cells were treated with 9-CRA, ATRA and MB either alone or in combination. Cells were grown until they formed visible colonies (21 d for LNCaP-P and LNCaP-N and 28 d for LNCaP-32). Medium was changed every 4 d. Colonies were fixed with formalin and stained with crystal violet as described in Materials and Methods. The numbers on the dishes correspond to the following concentrations of 9-CRA, ATRA and MB. 1 = control; 2: MB (0.5 nM); 3 = 9-CRA (0.5 µM); 4. ATRA (0.5 µM); 5 = MB+CRA; 6. MB+ATRA; 7 = CRA+ATRA (0.5 µM); 8 = MB = (1 nM). Note that the combined treatment is more potent and that the growth of LNCaP-32 cells is more profoundly inhibited.

**Figure 8:**
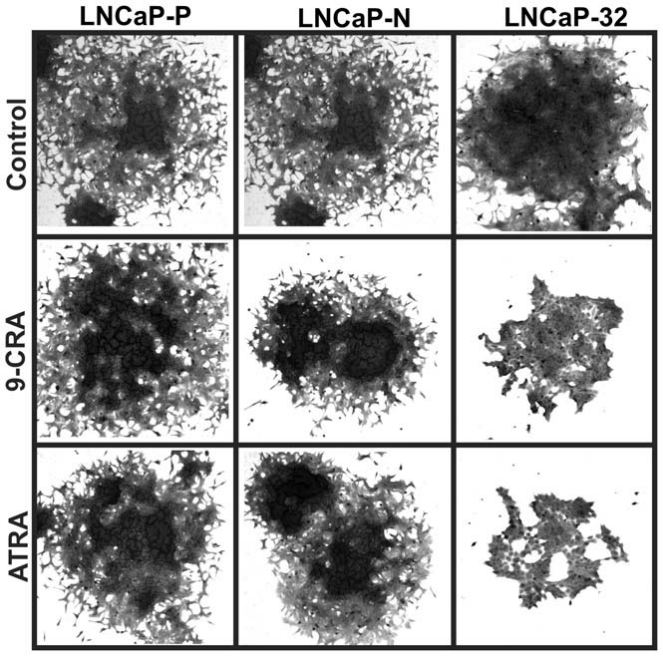
Retinoids affect the morphological phenotype of Cx32-expressing LNCaP cells. The morphological appearance of colonies of LNCaP-P, LNCaP-N and LNCaP-32 cells upon treatment with 9-CRA and ATRA is altered. Cx-null LNCaP-P and LNCaP-N cells form colonies of moderately packed cells with scattered edges whereas Cx32-expressing LNCaP-32 cells form colonies of compact cells with smooth edge. Note that upon treatment with 9-CRA and ATRA, LNCaP-P and LNCaP-N form colonies of more tightly packed cells with relatively smoother edges. Note also that these changes are profound in LNCaP-32 cells. For direct comparison of colony size and cell shape, all photographs were taken at the same magnification (40×). Colonies were fixed and photographed at day 21 (LNCaP-P and LNCaP-N) and day 28 d for LNCaP-32 cells.

To test if Cx32 expression potentiates the growth inhibitory effect of 9-CRA, ATRA and androgens, we measured cell growth by treating LNCaP-32, along with LNCaP-P and LNCaP-N cells, with various non-toxic concentrations of these agents. Cell growth was determined by the colony forming assay (Figures 6 and 7) and by counting the number of cells (Tables 3 and 4). As assessed visually by the size of the colonies, we found that the growth of LNCaP-32 cells was profoundly inhibited by MB, 9-CRA and ATRA whereas the growth of LNCaP-P and LNCaP-N cells was not substantially affected (Figure 6). We also found that while combined treatment with MB and 9-CRA or ATRA inhibited growth more drastically compared to treatment with either agent alone in LNCaP-P and LNCaP-N cells, these effects were more pronounced in LNCaP-32 cells (Figure 7). For example, in order to see visible colonies, dishes of LNCaP-32 cells treated with MB and 9-CRA or ATRA had to be fixed 10–13 days later compared with the dishes of LNCaP-P and LNCaP-N cells. The data shown in Figures 6 and 7 were substantiated by counting the number of cells in replicate cultures in two independent experiments (Table 3 and 4). For example, the growth of LNCaP-P and LNCaP-N cells was inhibited by only 20–25% upon treatment with 9-CRA, ATRA and MB whereas the growth of LNCaP-32 cells was inhibited by 40–55% (Table 3). Similarly, the growth of LNCaP-32 cells was more profoundly inhibited with the combined treatment with MB and 9-CRA or ATRA compared with LNCaP-P and LNCaP-N cells (Table 4). Moreover, in agreement with our previous findings [14], we found that LNCaP-32 cells formed more compact colonies with smooth edges compared with LNCaP-P and LNCaP-N cells which formed colonies with scattered edges (Figure 8). Furthermore, we also observed that 9-CRA and ATRA treated LNCaP-P and LNCaP-N cells became flatter and more tightly packed compared to untreated cells and these changes were more robustly pronounced in LNCaP-32 cells (Figure 8). Finally, the morphological changes similar to those observed upon treatment with 9-CRA and ATRA were not as pronounced when cells were treated with MB (not shown) although growth was inhibited.

**Table 3:** Cx32 expression sensitizes LNCaP cells to growth inhibitory effect of 9-CRA, ATRA and MB.

Treatment	LNCaP-P	LNCaP-N	LNCaP-32
**Expreiment #** 1			
Control	8.2±1.8 (100±22)	7.5±1.4 (100±19)	6.8±1.8 (100±26)
MB	6.4±1.4 (78±22)	6.1±1.2 (81±20)	3.2±0.8 ( 47±25 )
9-CRA	6.8±1.6 (83±23)	6.3±1.1 (84±17)	3.0±0.5 (44±17)
ATRA	6.2±1.3 (76±21)	5.9±1.3 (79±22)	2.9±0.6 (43±21)
**Experiment #** 2			
Control	7.8±1.1 (100±14)	8.1±1.0 (100±12)	7.2±1.3 (100±18)
MB	6.3±1.0 (81±16)	6.5±1.2 (80±18 )	3.3±0.6 (46±18)
9-CRA	6.5±0.9 (83±16)	6.7±1.5 (83±22)	3.1±0.4 (43±13)
ATRA	6.2±0.7 (79±11)	6.4±1.1 (79±17)	2.8±0.5 (39±18)

LNCaP-P, LNCaP-N and LNCaP-32 cells were seeded in 6-cm dishes in replicate (5×10^4^ cells/dish) and treated with 9-CRA (1 µM), ATRA (1 µM), and MB (2.5 nM). Cells were grown for 8 days with a medium change at day 5. Cells were trypsinized and counted as described in Materials and Methods. The values represent Mean number of cells per dish×10^5^±SE of the Mean. Values in the parentheses represent Means of % Growth ± SE of the Mean.

**Table 4:** Effect of 9-CRA, ATRA and MB on growth of Cx-null and Cx32-expressing LNCaP cells.

Treatment	LNCaP-P	LNCaP-N	LNCaP-32
**Expreiment #** 1			
Control	8.0±1.3 (100±16)	8.5±1.2 (100±14)	7.8±1.3 (100±17)
MB (0.5 µM)	6.6±1.2 (83±18)	6.7±1.4 (81±21)	3.9±0.9 ( 50±23 )
9-CRA	6.7±1.3 (84±19)	6.6±1.0 (84±15)	4.3±0.6 (55±14)
ATRA	6.5±1.5 (81±23)	6.1±1.2 (79±20)	4.1±0.4 (54±10)
MB+9-CRA	5.5±1.5 (69±27)	4.8±1.1 (56±21)	1.8±0.3 (23±17)
MB+ATRA	4.9±1.1 (61±22)	5.0±1.7 (59±21)	2.1±0.5 (27±24)
-CRA+ATRA	5.2±1.2 (65±23)	5.1±1.2 (64±21)	5.7±0.7 (73±12)
**Experiment #** 2			
Control	8.2.±1.3 (100±16)	8.3±1.2 (100±14)	7.6±1.1 (100±14)
MB	6.1±1.0 (74±16)	6.8±1.0 (82±15 )	3.1±0.4 (41±13)
9-CRA	6.0±0.9 (73±15)	6.4±1.3 (77±20)	3.5±0.3 (46±9)
ATRA	6.3±0.7 (77±11)	6.0±1.5 (72±25)	3.1±0.7 (41±23)
MB + 9-CRA	5.1±1.0 (62±20)	5.3±0.9 (64±17)	2.1±0.3 (28±14)
MB + ATRA	5.4±0.6 (66±12)	5.6±0.8 (67±14)	2.2±0.4 (29±18)
9-CRA + ATRA	4.9±0.8 (60±16)	4.9±0.6 (59±12)	4.7±0.5 (62±11)

LNCaP-P, LNCaP-N and LNCaP-32 cells were seeded in 6-cm dishes in replicate (5×10^4^ cells/dish) and treated with 9-CRA (0.5 µM), ATRA (0.5 µM), and MB (1 nM) either alone or in combination. Cells were grown for 11 days with a medium change at day 4 and 7. Cell growth was determined as in Table 3. The values represent Mean number of cells per dish×10^5^±SE of the Mean. Values in the parentheses represent Means of % Growth ± SE of the Mean.

## Discussion

Our findings demonstrate that retinoids enhance the expression level of Cx32 and formation of functional GJs in androgen-responsive human PCA cell line, LNCaP, which expresses retrovirally introduced Cx32. Earlier studies with these cells had shown that androgens enhanced GJ formation by controlling the expression level of Cx32 posttranslationally in a novel way — by rescuing the ERAD-targeted pool of Cx32 and rerouting it to the cell surface for GJ formation [17]. A key feature of our findings is that, apart from enhancing GJ assembly in normal medium, 9-CRA and ATRA also enhanced assembly by preventing degradation of Cx32, which is triggered upon androgen depletion in LNCaP-32 cells, independently of AR expression level or function. Only the assembly of Cx32 into GJs appeared to be significantly facilitated by 9-CRA and ATRA as neither the assembly nor degradation of adherens junction associated proteins, E-cadherin and α and β catenin, were significantly affected although the assembly of tight junction associated protein, occludin, appeared to be enhanced. The significance of these findings is further underscored by the fact that both androgens and retinoids have been documented to maintain the polarized and differentiated state of epithelial cells of normal prostate and prostatic tumors [19], [42]–[45], and all have been shown to have pleiotypic effects by acting as transcription factors [1], [3], [5], [46]. Our findings also showed that Cx32 expression potentiated the growth inhibitory effect of androgens and retinoids in LNCaP-32 cells, which indicates that their growth inhibitory and chemopreventive effects in prostate might be related to their ability to induce GJ formation.

Several independent lines of inquiry prompted us to undertake these studies. First, the expression of Cx32 had generally been found to coincide with the differentiated state of cells in the prostate as well as in other well-differentiated and polarized cells of several other organs [14], [38], [47]–[52]. Second, both androgens and retinoids had previously been shown to affect prostate morphogenesis and oncogenesis in animal models and in cell lines, including LNCaP [18], [19], [42]–[45], [53]–[56]. Third, previous studies had shown that retinoids enhanced GJ formation in several in vivo and in vitro model systems, indicating that their chemopreventive and pro-differentiating actions might be related to their ability to enhance GJ assembly [7], [25]–[27], [57]–[59]. Fourth, Cx32 has been documented to be a tumor suppressor in tissues in which it is expressed [60]–[62] and it seemed reasonable that its expression and assembly into GJs might be regulated by retinoids either alone or in conjunction with androgens.

How might retinoids enhance GJ assembly and prevent disassembly in LNCaP-32 cells? Our earlier studies with LNCaP-32 cells had shown that androgen-depletion attenuated GJ assembly by triggering the degradation of Cx32 by ERAD and not by modulating the transcription of the endogenous Cx32 gene or affecting Cx32 mRNA level driven by the retroviral promoter [17]. Because 9-CRA and ATRA neither induced the expression of the endogenous Cx32 in parental LNCaP and LNCaP-32 cells nor affected retroviral driven Cx32 mRNA transcripts, it seems likely that they enhanced GJ assembly by preventing the androgen-regulated pool of Cx32, posttranslationally, both under normal culture conditions as well as under androgen depleted condition [17]. It is well-established that AR is degraded upon androgen removal [63] and the expression level of AR in LNCaP cells has been known to be enhanced by 9-CRA and ATRA [30]. Our previous studies [17] had shown that AR-mediated signaling was the predominant factor in maintaining Cx32 expression level and preventing its degradation, posttranslationally, under androgen-depleted conditions in LNCaP-32 cells or in androgen-containing medium in the presence of anti-androgen, Casodex, which inhibits AR-function [64]. While our data showed that both 9-CRA and ATRA enhanced the expression level of AR under androgen-depleted conditions (Figure 4 A), GJ assembly was also robustly enhanced in the presence of Casodex despite low level of AR (Figure 4 B). It is possible that under androgen-depleted conditions, degradation of Cx32 is prevented by 9-CRA and ATRA by modulating the expression level of AR, leading to enhanced GJ assembly (Figure 4). However, our data also showed that when AR function was inhibited with anti-androgen, Casodex, androgen-mediated enhancement of Cx32 expression was annulled in normal serum as well as in androgen-depleted medium supplemented with MB, but the enhancement of GJ assembly mediated by 9-CRA and ATRA was not affected despite low AR expression. One possible explanation for these data is that 9-CRA and ATRA enhance GJ assembly by an AR-dependent mechanism under androgen-depleted conditions by rescuing the same pool of Cx32 which normally is ERAD targeted, yet activate another signaling pathway to enhance GJ assembly when AR function is inhibited by casodex under normal conditions. Further studies are required to explore this possibility.

Cadherins have previously been shown to facilitate the trafficking and assembly of Cxs into GJs and their loss has been shown to have the opposite effect on GJ assembly [32], [33], [37], [65]. However, our data documented that 9-CRA and ATRA had no significant effect on the degradation of adherens junction associated proteins E-cadherin or α and β catenins both under normal and androgen-depleted conditions (Figures 2 and 3). Therefore, it seems less likely that the degradation of Cx32, and GJs composed of it, was triggered indirectly by the loss of E-cadherin. Previous studies in other cells [29], [38], [39], and our earlier studies with LNCaP-32 cells [17], had shown that the trafficking of occludin to the cell surface and its detergent solubility was controlled by the assembly of Cx32 into GJs as androgen-depletion caused its internalization into intracellular stores [17]. In this regard, it is worth noticing that 9-CRA and ATRA increased both the total level of occludin as well as its detergent insolubility, although not robustly, which suggests that the assembly of Cx32 and occludin might be coordinately regulated by retinoids, and that this might be one of the mechanisms by which retinoids act as chemopreventive agents and maintain the polarized and differentiated state of epithelial cells. Further studies are required to substantiate this notion.

A salient feature of the data presented here is that the expression of Cx32 potentiated the growth inhibitory effect of androgens, 9-CRA and ATRA in LNCaP-32 cells as compared with their effect on LNCaP-P and LNCaP-N cells (Figure 6, Table 3). Moreover, combined treatment with the androgens and 9-CRA and ATRA was more potent than treatment with the either agent alone (Figure 7, Table 4). Treatment of LNCaP cells with androgens, 9-CRA and ATRA has had complex effects on growth, with some concentrations inhibiting growth and others enhancing growth [66]–[73]. In our studies, the concentrations of MB, 9-CRA and ATRA chosen were only marginally growth inhibitory to Cx-null LNCaP-P and LNCaP-N cells, but were profoundly growth inhibitory to Cx32-expressing LNCaP-32 cells. It is worth mentioning that in agreement with our earlier studies in other cell culture model systems [26], but in contrast to the complexity of the effect of MB, 9-CRA and ATRA on cell growth [66]–[73], these agents consistently enhanced the expression level of Cx32 and its assembly into GJs both in normal serum and under androgen-depleted condition in LNCaP-32 cells.

What might be the possible explanation for these findings? It is as yet unknown which signaling pathways are activated or suppressed upon formation and degradation of GJs [8], [10], [74] and how they are causally linked to the growth inhibitory effect [28], [58], [59]. Because androgens, 9-CRA and ATRA also inhibited the growth of Cx-null cells, and inhibition was potentiated upon expression of Cx32, it seems more likely that the formation of GJs either modulates a signaling pathway different from that activated by androgens and 9-CRA and ATRA or amplifies a pathway(s) activated by them or both. In previous studies we had shown that expression of Cx32 in LNCaP cells not only inhibited growth in vivo and in vitro but also induced differentiation as assessed by the ability to synthesize prostatic specific antigen, which is expressed by the well-differentiated luminal cells of the prostate [14]. Moreover, these studies also showed that serial propagation of Cx32-expressing LNCaP sub-clones caused selection of cells in which the capacity to assemble GJs was lost as only few sub-clones could be established that retained the ability to form GJs and in which the growth suppressing effect of junction formation was compensated by other growth-modulatory mechanisms [14]. In this regard, it is noteworthy that transient expression of Cx43 in LNCaP cells via adenoviruses sensitizes these cells to apoptosis induced by tumor necrosis factor α, TRAIL, and anti-Fas antibodies to which Cx-null LNCaP cells are resistant. Because formation of functional GJs was required for sensitization and Cx expression had no effect on TNF-α receptor number, Wang et al. have proposed that transmission of some small molecules from cell-to-cell acted as an apoptotic trigger [75]. It is at present difficult to envisage how chemopreventive, pro-differentiating and growth-inhibitory effects of 9-CRA and ATRA are tied to the formation of GJs as the expression of several classes of genes has been shown to be altered by them [11], [12], [28], [59], [76], [77].

The incidence of PCA escalates dramatically at ages when men confront other competing causes of mortality. Prostate cancer is a slow growing tumor with a long latency period and while the incidence of histologically detectable PCA is high, the incidence of clinically detectable disease is low and is influenced by dietary factors [19], [71], [78]. Moreover, this long latency period affords opportunities for intervention with therapies that are designed to delay disease initiation and/or progression. Although retinoids have been considered as one of the candidate dietary factors in controlling PCA progression, epidemiological data have been controversial as both suppressive and stimulatory effects have been reported [24], [79]–[81]. Androgens and retinoids are required for the maintenance of normal healthy prostate epithelium as in their absence glandular atrophy of the prostate ensues due to massive apoptosis of luminal epithelial cells [18], [82]. Although retinoids have been known to be chemopreventive agents for decades and have been used as differentiating agents, their use in human clinical trials has been hampered due to pleiotypic and toxic effects [24], [83], [84]. Thus, the role of androgens and retinoids in suppressing and facilitating PCA progression as well as in maintaining the differentiated state of epithelial cells of normal prostate and prostate tumors has remained enigmatic [18], [19], [79], [83], [85]. In the light of the effects of androgens and 9-CRA and ATRA on junction formation and growth, and because of the potential interactions among AR and RXR receptor pathways, an investigation into their additive and/or synergistic effects on the assembly and disassembly of Cx32 into GJs might shed light on the mechanism by which retinoids and androgens affect growth, differentiation and apoptosis of normal and PCA cells.
